# Whole Genome Sequencing Reveals High Genetic Diversity, Diverse Repertoire of Virulence-Associated Genes and Limited Antibiotic Resistance Genes among Commensal *Escherichia coli* from Food Animals in Uganda

**DOI:** 10.3390/microorganisms11081868

**Published:** 2023-07-25

**Authors:** Denis K. Byarugaba, Godfrey Wokorach, Stephen Alafi, Bernard Erima, Florence Najjuka, Edison A. Mworozi, Hannah Kibuuka, Fred Wabwire-Mangen

**Affiliations:** 1 Makerere University Walter Reed Project, Kampala P.O. Box 16524, Uganda; wokosiki@gmail.com (G.W.); salafi@muwrp.org (S.A.); berima@muwrp.org (B.E.); hkibuuka@muwrp.org (H.K.); fwabwire@musph.ac.ug (F.W.-M.); 2 College of Veterinary Medicine, Makerere University, Kampala P.O. Box 7062, Uganda; 3 Gulu University Multifunctional Research Laboratories, Gulu P.O. Box 166, Uganda; 4 College of Health Sciences, Makerere University, Kampala P.O. Box 7062, Uganda

**Keywords:** MLST, genetic diversity, commensals, virulence genes, Shiga toxin

## Abstract

Commensal *Escherichia coli* with broad repertoire of virulence and antimicrobial resistance (AMR) genes pose serious public health risks as reservoirs of AMR and virulence. This study undertook whole genome characterization of commensal *E. coli* from food-producing animals in Uganda to investigate their genome variability (resistome and virulome). We established that the *E. coli* had high genomic diversity with 38 sequence types, 24 FimH types, and 33 O-antigen serotypes randomly distributed within three phylogroups (A, B1, and E). A greater proportion (≥93.65%) of the *E. coli* were resistant to amoxicillin/clavulanate and ampicillin antibiotics. The isolates were AmpC beta-lactamase producers dominated by *bla*_EC-15_ (71.88%) and *tet(A)* (20.31%) antimicrobial resistant genes besides a diverse armory of virulence-associated genes in the class of exotoxin, adhesins, iron uptake, and serine protease autotransporters which varied by host species. Cattle were found to be the major source of *E. coli* carrying Shiga toxin genes, whereas swine was the main source of *E. coli* carrying colicin-like *Usp* toxin gene. The study underscores the importance of livestock as the carrier of *E. coli* with antimicrobial resistance and a large repertoire of virulence traits with a potential of causing disease in animals and humans by acquiring more genetic traits.

## 1. Introduction

*Escherichia coli* is a very versatile bacterium that is known to survive in different niches including in warm blooded mammals and the environment due to its genomic plasticity. It comprises pathogenic and non-pathogenic strains which sometimes co-exist in specific niches such as the gut. To survive in the gut, they must be able to withstand the conditions therein, including the assault by the host gut immune system and other chemicals that reach the gut, such as antimicrobial agents. Those that are susceptible to the host immune system and antibacterial agents will be eliminated. The gut commensal bacteria must therefore possess minimal protective attributes such as virulence traits and antimicrobial resistance to the antimicrobials they are frequently exposed to. These traits enable the bacteria to survive the assault and competition with other microbes within those systems [1].

*E. coli* are generally known to belong to A, B1, B2, C, D, E, and F phylogenetic groups. The phylogroup classification is based on the presence or absence of four genes (*chuA*, *yjaA*, TspE4.C2, and *arpA*) [2]. Phylogroups A and B2 are predominant among animals, while phylogroup B2 and D are responsible for most infections in humans [3]. The pathogenic strains are highly adapted, with specific virulence attributes which enable them to cause disease in different parts of the body, which has come to define their pathotypes, namely, intestinal (or diarrheagenic) pathogenic *E. coli* (IPEC) and extra-intestinal pathogenic *E. coli* (*ExPEC*). There are several pathotypes based on the disease and/or pathogenesis, including uropathogenic *E. coli* (UPEC), neonatal meningitis *E. coli* (NMEC, meningitis-associated *E. coli* (MAEC), avian pathogenic *E. coli* (APEC), enteropathogenic *E. coli* (EPEC), Shiga toxin-producing *E. coli* (STEC); enterohemorrhagic *E. coli* (EHEC); enterotoxigenic *E. coli* (ETEC); enteroinvasive *E. coli* (EIEC); and enteroaggregative *E. coli* (EAEC), diffuse adhering *E. coli* (DAEC), adherent-invasive *E. coli* (AIEC), sepsis-associated *E. coli* (SEPEC) and unspecified ExPEC, as previously described [4].

Several of these pathotypes, such as APEC, EPEC, EHEC, and ETEC, cause disease in animals with similar virulence factors as in humans. The EHEC strains of *E. coli* produce “Shiga toxins”, encoded by the *stx1* and/or *stx2* gene common to all EHEC bacteria, and are also pathogenic for animals and humans [5]. The differentiation between commensal *E. coli* and others has been challenged because of the frequent genomic alterations and genetic exchanges within and between strains, where non-pathogenic strains acquire virulent genes gradually gaining specific traits for specific pathotypes [6].

*E. coli* has been found to be the largest contributor to the global burden of antimicrobial resistance causing death in humans [7]. For a long time, these bacteria have been exposed to and have become used to antimicrobial agents. The antimicrobial exposure exerted selective pressure that led to the emergence of *E. coli* strains that have acquired resistance to multiple antibiotics and a diverse array of virulence factors that have been successfully disseminated globally. Strains that produce extended-spectrum β-lactamases (ESBL), in particular, often result in longer hospitalization, higher morbidity, and increased health-care costs [8,9]. *E. coli* have a broad habitat, including the gastrointestinal tract of most warm-blooded animals as commensals, soil, water, and sewage, where they are frequently exposed to antibiotics and other chemicals that have made them develop survival mechanisms. They are, therefore, frequently used as resistance indicators [10]. A few of the *E. coli* that cause intestinal and extraintestinal infections have been reported both in animals and humans.

A lot of interest has been focused on the burden of multi-drug resistant (MDR) strains in humans [11] and their potential sources, such as environmental and animal reservoirs. However, less consideration has been given to humans as potential reservoirs of MDR for animals and the environment [12]. Besides the pathogenic *E. coli*, commensal bacterial are known to harbor and exchange resistance and virulence loci and can provide a reservoir of resistance genes, which may be transferred between bacterial species, including pathogens [13]. The resistance genes and other fitness attributes are successfully disseminated and shared among *E. coli* strains within the ecological environment where they survive. The sharing of genetic information among *E. coli* strains is a threat to public health, animal health, and environmental health from one health perspective [14]. Several factors including antibiotic use, limited infection and prevention, and biosecurity on farms, both in animals and humans, are the major drivers that enhance the development, persistence, and dissemination of resistant organisms [15]. The predominant use of beta-lactams and tetracyclines antibiotics to treat animals in Uganda has driven the increased resistance to the drugs, which is presenting a serious clinical challenge. In particular, β-lactamases of class C are widely distributed on the chromosomes of many Gram-negative species typically annotated as *ampC*, which under normal conditions are not expressed but high-level expression is often induced by mutation or induction by specific β-lactams that lead to clinical resistance [16].

Human studies have more frequently reported highly successful globally disseminated pandemic high-risk clones, such as ST-131, ST-648, ST-10, and others [17], which are responsible for many health care associated and community infections, and treatment failures. Many of these clones are increasingly being reported in animals and the environment, which enhances the hypothesis that animals may act as reservoirs of *E. coli* strains pathogenic to human [18]. This study aimed to establish the genomic diversity and existence of high-risk clones, resistome, and virulome within *E. coli* isolates from livestock in Uganda.

## 2. Materials and Methods

### 2.1. Sample Collection

A total of 729 samples were collected from: cattle (n = 225), swine (n = 245), and poultry (n = 257) from September 2020 to April 2021, using a series of cross-sectional studies at swine slaughter places, cattle abattoirs, and live poultry markets in Kampala and Wakiso districts. For cattle and swine, fecal samples were collected, while for poultry, fecal droppings were collected in 275 mL of peptone water.

### 2.2. Bacteria Isolation

Samples were suspended in peptone water, incubated overnight, and inoculated directly onto Xylose/Lysine/Deoxycholate (XLD) selective agar and incubated at 37 °C overnight. Yellow colonies that were catalase positive and oxidase negative were confirmed as *E. coli* with API-20E kits (bioMérieux, Inc., 100 Rodolphe Street, Durham, NC, USA). Isolates were subjected to antibiotic susceptibility testing by standard disk diffusion test according to CLSI [19]. Multi-drug resistant isolates were selected for whole genome sequencing and sent to Walter Reed Army Institute of Research (WRAIR)’s Multi-Drug Resistant Organism Repository and Surveillance Network (MRSN) in the USA.

### 2.3. DNA Library Preparation

A total of 63 multidrug resistant isolates were selected and grown on MacConkey agar for 48 h at 37 °C. The isolates were collected in 1.5 mL tubes and extraction of DNA was completed with DNeasy UltraClean microbial kit (Qiagen, Germantown, MD, USA). DNA libraries were prepared using Kapa HyperPlus kit (Roche Diagnostics, Indianapolis, IN, USA). Libraries’ concentrations were evaluated in a CFX96 real-time iCycler machine (Bio-Rad, Hercules, CA, USA) using KAPA™ Library Quantification Kits (Roche Diagnostics). Paired-end sequencing was performed at MRSN on the Illumina NextSeq machine (Illumina, Inc., San Diego, CA, USA).

### 2.4. Sequence Analysis

Removal of sequence adapters from raw reads was performed using Btrims [20]. Denovo assembly of raw reads was performed with Newbler (v2.9) [21]. Taxonomic assignment of the isolates using assembled genome sequences was performed by matching against typed reference genomes in the Genome Taxonomy Database (GTDB). A close match with an Average Nucleotide Identity (ANI) value ≥ 95% was used to infer the species of the isolates. High-quality SNPs were generated from concatenated alignment of core genome genes which was then used to build a phylogenetic tree using CSI Phylogeny. The tree was imported into Interactive Tree of Life (https://itol.embl.de/, accessed on 1 March 2023) for viewing and annotation. The in silico ClermonTyping was used to group the isolates into different phylogroups [22]. Assigning the isolates into different Multilocus Sequence Types (MLST) was performed by comparison to sequences available in MLST v2.0 database [23]. SeqSphere+ was used to generate the minimum spanning tree from *E. coli* isolates based on the MLST scheme [24]. Serotype prediction was performed using SerotypeFinder v2.0 database [25] and FimType prediction was performed using FimTyper v1.0 database [26]. mlplasmids v2.1.0 was used to identify contigs that were of plasmid origin with posterior probability set at 0.9 and minimum contig length of 1000 bp [27]. Contigs identified as plasmids were further confirmed using NCBI nucleotide blast. Antibiotic-resistant genes were identified and confirmed among the isolates using ResFinder 4.1, AMRFinder, and CARD databases within the plasmid and chromosomal DNA [28]. VirulenceFinder v2.0 was used to predict the occurrence of various virulent determinants among the isolates [29]. For all the software used, default parameters were considered unless otherwise indicated.

## 3. Results

### 3.1. Antimicrobial Susceptibility Test

The antimicrobial susceptibility test was performed on nine antibiotics. Generally, there were significantly high levels of antibiotic resistance observed with amoxicillin/clavulanate and ampicillin (≥93.65%) antibiotics. Antibiotics with moderate levels of resistance were ceftazidime (47.62%), ciprofloxacin (57.14%), gentamicin (49.21%), and trimethoprim/sulfamethoxazole (53.97%), whereas the majority of isolates were susceptible to chloramphenicol (79.37%), cefotaxime (63.49%), and meropenem (80.95%) antibiotics. Resistance to amoxicillin/clavulanate was significantly high in *E. coli* isolated from chickens, swine, and cattle, whereas resistance to ampicillin antibiotics was particularly high among *E. coli* isolated from cattle and swine. All *E. coli* isolated from chicken were found to be resistant to ciprofloxacin. Resistance to ciprofloxacin was equally high among *E. coli* isolated from swine.

### 3.2. Phylogroups, STs, FimH Types, and Serogroup of the E. coli Isolates

**Figure 1:**
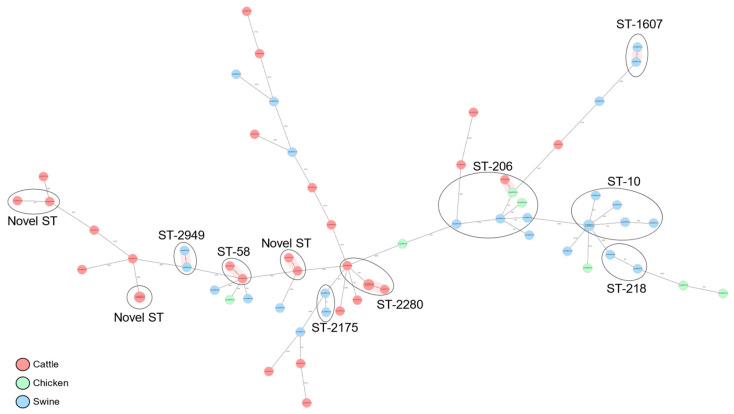
cgMLST-based minimum spanning tree of 63 commensal *E. coli* isolates recovered from cattle, swine, and chicken slaughter places in Kampala. Isolates belonging to the same dominant sequence types (ST) are circled and labeled and the hosts of origin are shown in different colors.

Phylogroups B1 (57.81%) and A (31.25%) were most commonly associated with the three host species, while phylogroups E, C, and D were marginally detected. Phylogroups’ compositions were largely dependent on the animal species. For example, phylogroup A was principally isolated from swine and chicken, whereas phylogroup B1 was predominantly isolated from cattle. A total of 39 known MLST groups were detected among the 63 isolates (Figure 1). ST10 (9.38%), ST-206 (9.38%), and ST-2280 (6.25%) were the commonest *E. coli* sequence types associated with the animals.

**Figure 2:**
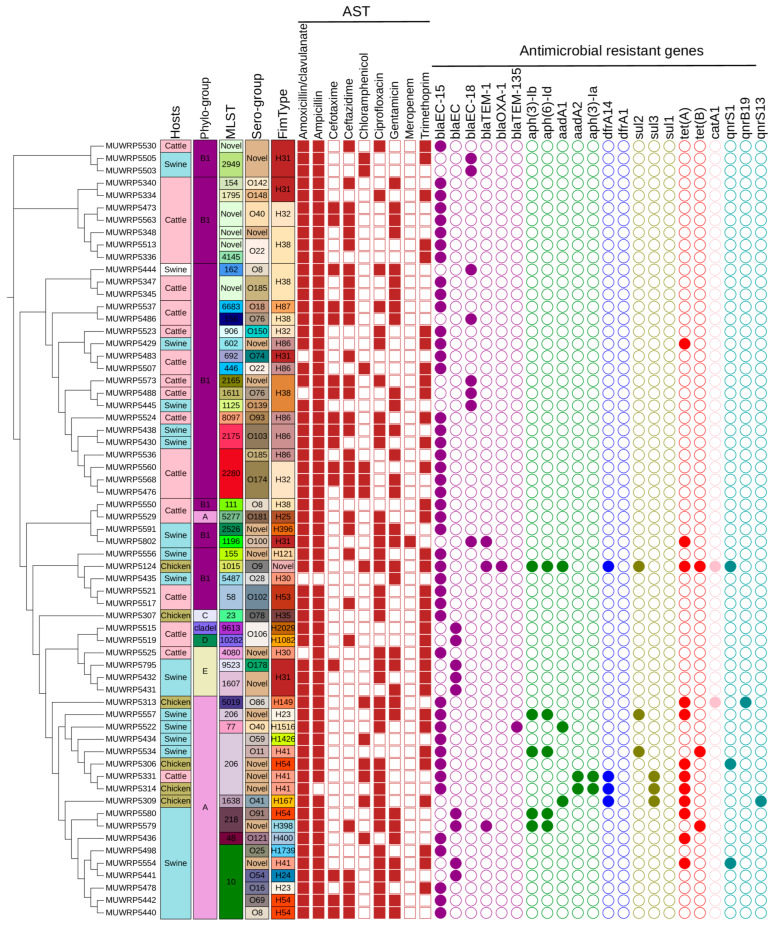
SNP-based phylogenetic tree of 63 *E. coli* strains analyzed in this study characterized by phylogroup, sequence type (ST), isolation sources, and sample location with the corresponding antimicrobial resistance genes (absent, open circle; present, filled colored circle according to the class of AMR genes).

Most of the MLST groups were detected only marginally, whereas a significant proportion of the *E. coli* isolates (10.94%) were from novel STs. The distribution of the different MLST groups were determined by the different phylogroups and animal species. The ST-206 and ST-10 were essentially detected only in phylogroup A (Figure 2). Whereas ST-206 was distributed in all three animal species, ST-10 was restricted within the swine host. Other STs associated with phylogroup A were ST-218, ST-48, ST-484, ST-5019, ST-5277, and ST-77. All novel STs and ST-2280 uniquely cluster within phylogroup B1/cattle host (Figure 2).

The *E. coli* clustered into 24 different FimH types. FimH31, H38, H32, and H38 were the most common (Figure 2). The majority of fimtypes were marginally detected and not present in more than one isolate. Fim H38 and H32 were majorly detected in *E. coli* isolated from cattle which reflects their host dependency. Meanwhile, H31 and H86 fimtypes were restricted within cattle and swine hosts. Only H41 was found to have a broader host range as it occurs in all three animal host species. Serogroups O174, O185, O22, and O76 were detected only in cattle and were the commonest, whereas other serogroups were only marginally detected. A greater proportion of the isolate could not be typed into known serogroups (Figure 2).

### 3.3. Presence of Antimicrobial Resistant Genes

A limited occurrence of different antimicrobial-resistant genes within the genomes of the *E. coli* isolates was observed. More than half of the isolates (76.19%) had only one antimicrobial-resistant gene within their genome. The most-frequent antibiotic-resistant gene detected was the *bla*_EC-15_, which occurs among (71.88%) of the *E. coli* isolates. The other beta-lactam genes detected were *bla*_EC_ (14.06%), *bla*_EC-18_ (12.50%), and *bla*_TEM-1_ (6.25%). Tetracycline-resistant, *tet(A)* gene was detected among 20.31% of isolates. Only a limited proportion (4.69%) of isolates were found to carry the *tet(B)* gene. The quinolone group of antibiotics occurs only within a limited number of *E. coli* isolates (n = 5). Among the quinolone genes detected were the *qnrS1* (4.36%), *qnrB19* (1.56%), and *qnrS13* (1.56%). The folate pathway antagonist genes detected were *dfrA14* (6.25%), *sul2* (6.25%), *sul3* (4.6%), and *sul1* (1.56%). The aph-(3)-Ib and aph-(6)-Id aminoglycoside-resistant genes were both detected at a frequency of 9.38%. Other rare aminoglycoside genes were *aadA1* (6.25%), *aadA2* (3.13%), and *aph(3)-Ia* (3.13%). The O9-serogroup isolate was associated with multiple antibiotic-resistant genes including *bla*_EC-15_, *bla*_TEM-1_, (aph (3)-Ib, aph (6)-Id), sul2, *tet*(A), catA1, and qnrS1. The *bla*_EC-15_ and tet(A) genes were predominantly located within phylogroups (B1 and A), whereas the *bla*_EC_ gene was restricted among phylogroups (B1 and E). The aminoglycoside genes, aph (3)-Ib and aph (6)-Id, were associated with phylogroup A.

**Figure 3:**
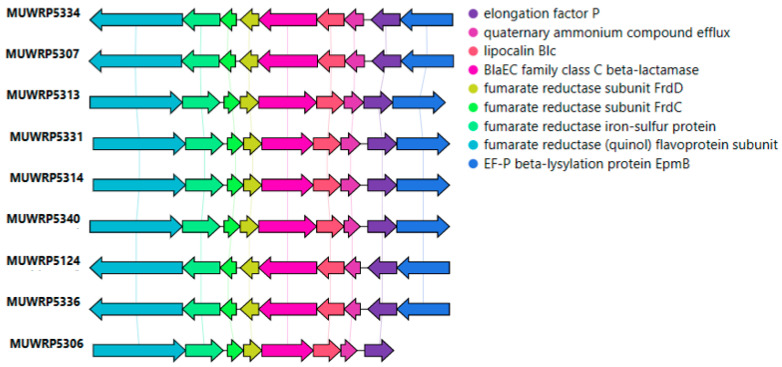
Gene arrangement around *bla_EC-15_* resistant gene.

None of the *bla*_EC-15_ genes were located within the plasmid. However, the *bla*_EC-15_ genes were bordered by the fumarate reductase gene cluster upstream and the lipocalin (Figure 3). The *bla*_EC-15_ gene was also in close association with the gene for quaternary ammonium compound efflux (Figure 3). Only two isolates were detected with plasmid contigs carrying antimicrobial-resistant genes. The isolate MUWRP5802 was found to have a plasmid contig with *blaTEM-1B* gene. The second isolate MUWRP5306 had a plasmid fragment that carries *tet(A)* and *qnrS1* antimicrobial resistant genes.

### 3.4. Characteristics of Virulence Determinants among the E. coli Isolates

**Figure 4:**
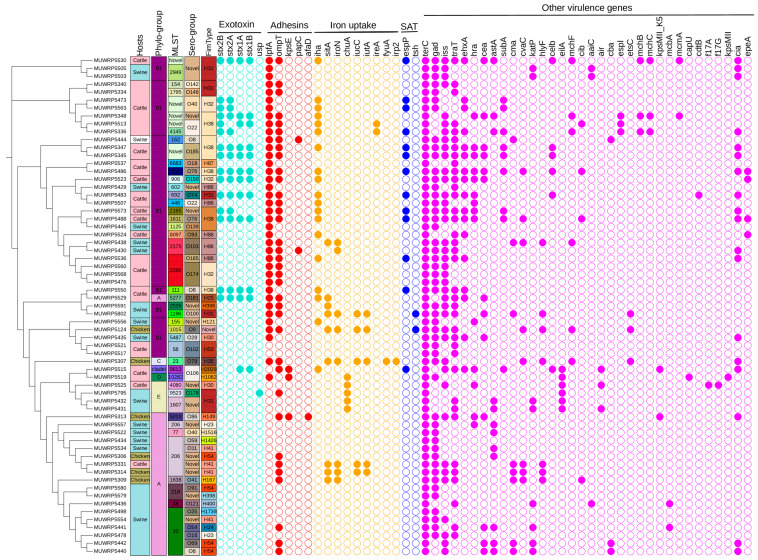
A SNP-based phylogenetic tree showing the distribution of virulent genes by phylogroup, sequence type (ST), serogroup, fimtype, and host (absent, open circle; present, filled colored circle according to the virulent genes).

The virulent gene classes detected were those belonging to exotoxin, adhesins, iron uptake, serine protease autotransporters (SAT), and more. The distribution of the different virulent genes largely depended on the host species, phylogroups, and serogroups (Figure 4). A predominantly high proportion of the virulence-associated genes was detected from *E. coli* isolated from cattle compared to those isolated from swine and chicken. Two classes of exotoxin virulent genes were detected: the colicin-like *Usp* toxin gene was rare and occurred within *E. coli* isolated from swine (phylogroup E and serogroup-O178). Meanwhile, cattle (phylogroup B1) were the major source of *E. coli* carrying Shiga toxin genes. The stx2B (23.44%) and stx2A (21.88%) were the most common Shiga toxin genes carried by most *E. coli* isolated from cattle (Figure 4). Additionally, phylogroup B1 was associated with many different types of adhesins genes (Figure 4). For example, the adhesin gene *lpfA* for long polar fimbria protein occurs in most of phylogroup B1, except those of serogroup (O102). Among the genes that regulate iron uptake, the *ireA* gene was limited to two isolates of phylogroup B1 (serogroup O22), whereas the *iha* gene was widely distributed among isolates of phylogroup B1. Another iron uptake gene, *chuA*, had limited occurrence in isolates of phylogroup E. Other virulence genes found exclusively among phylogroup E were *eilA* and *air* gene. The only members of the serine protease autotransporters gene family detected were *espP* and *Tsh* genes. The *espP* gene occurs mainly within cattle, whereas the *Tsh* gene was from *E. coli* isolated from chicken. Swine was the major carrier of *E. coli* isolates with *katP* gene for plasmid-encoded catalase-peroxidase protein and was encoded within the plasmid. Overall, most of the virulence-associated genes were detected at moderate proportion (20%-29%), whereas others, such as *terC*, *gad*, *iss*, *lpfA*, *traT*, and *ompT*, were very common (>55%) among *E. coli* from animal sources (Figure 4). The *ompT* and *hlyF* genes were found to occur in the same plasmid in six isolates. Additionally, *afaD* virulence gene was detected in only one isolate (MUWRP5313) and was encoded by plasmid.

## 4. Discussion

The current study examined the genomic diversity of commensal *E. coli* in view of their resistome and virulome, which may be of relevance to transmission of these pathogenic strains. Many studies have tended to focus on pathogens, with limited focus on non-pathogenic bacteria that are regularly excluded from surveillance programs, yet may serve as reservoirs of AMR in the environment and the food chains, underscoring the need for more comprehensive analyses and monitoring of food environmental and animal reservoirs of AMR [30,31]. With the threat of antimicrobial resistance and untreatable infections as a big global concern, the role of animals and environment reservoirs in sustaining and disseminating AMR can no longer be ignored, as recent evidence suggests that AMR genes in animal and environmental bacteria can be rapidly acquired by human-associated and pathogenic bacteria [32,33,34].

The commensal isolates in this study showed a high genetic diversity with as many sequence types as the isolates themselves. This phenomenon of high genomic plasticity within *E. coli* has been reported several times in most parts of the world, including Uganda [35]. The plasticity seen in this species is likely due to horizontal gene transfer, gene loss, as well as other genomic modifications, such as DNA rearrangements and point mutations, which constantly alter the genome content and thus the fitness and competitiveness of individual variants in certain niches [36]. We identified potential zoonotic sequence types, especially ST-10, which was only in isolates from swine and not the other species. ST-10 *E. coli* is among the international, globally disseminated high-risk pandemic clones that have been frequently incriminated in several human ExPEC infections. This ST clone has been reported in synanthropic animals carrying several resistance genes to antibiotics frequently used in humans [37]. Similarly, this sequence type has been common in food-producing animals [38,39], consistent with our findings. The isolates belonged to two main phylogroups, A and B1, unlike the predominance of B2 which is commonly reported in human pathogenic *E. coli*, especially the ExPEC but also those of intestinal disease agents. These findings are consistent with other studies [18,40,41] that have reported predominance of phylogroups A and B1 in food animals.

While the isolates in the current study demonstrated carriage of a limited number of antimicrobial resistance genes, they were all AmpC beta-lactamase producers carrying mainly β-lactamase class *bla*_EC_ genes, with 71.88% carrying the *bla*_EC-15_ gene. Similar findings have been reported [18]. The class C β-lactamases comprise the second most abundant group of enzymes and are found solely in Gram-negative bacteria, especially members of the *Enterobacteriaceae* [42]. Although they were originally identified as chromosomally encoded, more recently, genes encoding class C enzymes have been found mobilized on plasmids in the *Enterobacteriaceae* [43]. A smaller number of our isolates carried other resistances against tetracyclines *tet(A)* and *tet(B)* genes; quinolone resistance genes *qnrS1*, *qnrB19*, and *qnrS13*; as well as a few isolates with resistance genes for folate pathway antagonist genes and aminoglycoside-resistant genes. Resistance often follows antibiotic use patterns in animal populations [44]. The main antibiotics used in animal production in Uganda include penicillins, tetracylines, and sulphonamides [15,45,46,47].

A broad repertoire of virulence genes with invasion/adherence, exotoxin, and siderophore factors among the isolates obtained was demonstrated. Enteric *E.coli* consists of commensals that are not associated with disease but could cause disease under certain circumstances. To survive in the gut, *E. coli* strains frequently carry and are capable of toxin secretion, aggregative colonization, and multiplying in the gastrointestinal tract, and with the acquisition of additional genes could cause damage to different environments through the adaptation of key genetic elements, resulting in the formation of new pathotypes [42].

Most of the isolates had the *terC*, *gad*, *iss*, *lpfA*, and *ompT* virulence genes commonly found across the different animal hosts while the Shiga toxin genes (stx2B, stx2A, Stx1A, stx1B) occur mainly among *E. coli* of cattle origin. All isolates had the *terC* gene, which is part of the tellurite resistance gene operon (ter) that is known to exist widely and in many bacteria, particularly pathogenic species. The *terC* is part of the *ter* operon that is known to confer resistance to the oxyanion form of the rare nonessential trace element tellurium, namely, tellurite oxide (TeO_3_^−2^), and is implicated in tellurite resistance, phage inhibition, colicine resistance, and pathogenicity [48]. It has been said that the *ter* operon is required for fitness to survive in the gut where colicins from other competing bacteria and host antimicrobial peptides limit their survival. In *Klebsiella pnemoniae*, the *terC* has been reported to offer tolerance to ofloxacin, polymyxin B, and cetylpyridinium chloride as well [49]. The *ter* operon has also been demonstrated to be associated with stress tolerance factor and enhancing fitness in the gut, particularly against stress induced by the indigenous gut microbiota during colonization [50]. This confirms our observation that commensal bacteria need these anti-stress genes for survival within the microbial community in the gut. Similarly, we found most isolates in possession of the *gad* gene which encodes the glutamate decarboxylase pathway (GDP), which is a major acid resistance mechanism enabling microorganisms’ survival in low pH environments. The glutamate-dependent acid resistance system is said to be the most potent acid resistance system in commensal and pathogenic *Escherichia coli*. These gut bacteria need these traits to overcome the acidic environment during transit through the host stomach to successfully colonize the gut.

The Isolates had several other genes directly involved in pathogenicity. The Shiga toxin virulence genes, in particular, mediate the production of Shiga toxin among the enteric pathogenic *E. coli* strains (the Shiga toxin-producing *Escherichia coli*) which causes hemorrhagic colitis and hemolytic uremic syndrome [51]. Possession of the *stx* genes by animal *E.coli* does not necessarily indicate that they cause disease. It has been suggested that ruminants in particular do not have vascular globotriaosylceramide receptors where Shiga toxin binds and are always asymptomatic [52]. However, the ruminants can easily shed Shiga toxin producing *E. coli* in their feces and are considered reservoirs for Shiga toxin producing *E.coli* for humans.

The similarity in virulence factors between human ExPEC and *E. coli* from animal sources highlights the role of livestock in perpetuating infections in humans, including Shiga toxin-producing *E. coli* strain as reservoirs. While commensal *E. coli* commonly reside in human and animal gut without causing disease, they may occasionally cause opportunistic infections when the host immunity is impaired or injured. Their ability to acquire and transmit virulence and AMR genes within the gut both to non-virulent bacteria and pathogenic ones poses a serious threat that needs routine monitoring. They have been particularly found to be efficient in acquiring and transmitting these genes through mobile genetic elements such as transposons and plasmids [53].

## 5. Conclusions

Taken together, this study established the convergence of a limited resistome and a wide virulome among commensal AmpC beta-lactamase producing *E. coli* isolates in livestock in the country with a limited number of the international high-risk clones of pandemic importance. These clones have the potential to share both virulence and/or resistance genes to pathogenic *E. coli* or other pathogenic bacteria within the gastroenteric system.

## Data Availability

The nucleotide sequences were submitted to the NCBI database and are available under Bioproject ID: PRJNA977092.
